# A comprehensive strategy for exploring corrosion in iron-based artefacts through advanced Multiscale X-ray Microscopy

**DOI:** 10.1038/s41598-022-10151-w

**Published:** 2022-04-12

**Authors:** Martina Bernabale, Flavio Cognigni, Lorenzo Nigro, Marco Rossi, Tilde de Caro, Caterina De Vito

**Affiliations:** 1 Department of Earth Sciences, Sapienza University of Rome, P.le Aldo Moro 5, 00185 Rome, Italy; 2 Department of Basic and Applied Sciences for Engineering (SBAI), Sapienza University of Rome, Via Antonio Scarpa 14, 00161 Rome, Italy; 3 Department Italian Institute of Oriental Studies-ISO, Sapienza University of Rome, Circonvallazione Tiburtina 4, 00185 Rome, Italy; 4 Istituto per lo Studio dei Materiali Nanostrutturati-National Research Council (ISMN-CNR), Rome, Italy

**Keywords:** Chemistry, Engineering, Materials science, Nanoscience and technology, Physics

## Abstract

The best strategy to tackle complexity when analyzing corrosion in iron artefacts is to combine different analytical methods. Traditional techniques provide effective means to identify the chemistry and mineralogy of corrosion products. Nevertheless, a further step is necessary to upgrade the understanding of the corrosion evolution in three dimensions. In this regard, Multiscale X-ray Microscopy (XRM) enables multi-length scale visualization of the whole object and provides the spatial distribution of corrosion phases. Herein, we propose an integrated workflow to explore corrosion mechanisms in an iron-nail from Motya (Italy) through destructive and non-destructive techniques, which permit the extraction of the maximum information with the minimum sampling. The results reveal the internal structure of the artefact and the structural discontinuities which lead the corrosion, highlighting the compositional differences between the tip and the head of the iron nail.

## Introduction

One of the main challenges in Cultural Heritage is the inspection of inner and hidden parts of an artefact, that may provide crucial information with minimal sample processing. Invasive techniques are often the only way to explore the original alloys of complex objects from rim to core and the corrosion process when it occurs in depth^1–4^.

In particular, archaeological iron artefacts undergo corrosion phenomena, resulting in the loss of the metal core, which leads to the loss of information about the function of the object and the forging processing. The corrosion of iron consists of a stratification in two distinct zones, before reaching the inner metal core, if still present. The first region after the metal core, the so-called dense product layer (DPL), is constituted by iron oxides and oxyhydroxides and appears relatively dense. The second one, *i.e.*, transformed medium (TM), is composed of iron oxyhydroxides and typical minerals of soil^5^.

The past five decades has witnessed the emergence of many works in which various strategies are proposed to explore the layered corrosion system of iron artefacts from different environments through cross-sections preparation^5–7^. This approach is reliable, but the archaeological artefacts are unique pieces and cannot always be sacrificed. Furthermore, when allowed, sampling must be minimal and must not compromise the functionality and aesthetics of the object. Therefore, if the metal core is hidden deep, there is a risk that it will not be detected and studied.

In the last few years, X-ray imaging, i.e, radiography and tomography, has been exploited in archaeometry to investigate depth corrosion, volumes, morphology and to detect cracks and other structural defects non-destructively and in three dimensions (3D)^8–20^. X-ray Microscopy **(**XRM) had been already used in a huge variety of materials applications, such as lithium-ion batteries, aerospace, additive manufacturing, electronics and semiconductors engineering^21,22^ due to its capability to investigate specimens at their microstructural level with resolution down to the micro- and nano-meter scale in a completely non-invasive way, allowing to understand the failure mechanisms and the properties of materials^23^. For such reasons, this technique is especially well suited to the inspection of the internal structure of stratified archaeological objects. The major constraint arises from the impossibility to obtain chemical information on the sample. Because of this, “*correlative microscopy*” combines a variety of microscopy techniques to access a much larger range of information, such as microstructure, mineralogy and chemistry about a specific region of interest (ROI) of the sample^17^. This would not be possible by using a single instrument. Nevertheless, this requires the 2D/3D spatial registration of multiple techniques on the same ROI. Therefore, if the latter is located within a non-expendable zone of the artefact, this approach cannot be achieved without permanently damaging the sample.

In this work, we present a characterization workflow in which non-invasive and invasive techniques are combined to explore the corrosion system of an archaeological iron nail from the archaeological site of Motya (Sicily-Italy).

The iron nail (IV century BC) was unearthed in the US 1210 of the Area F, the so-called “Western Fortress”, during the excavations carried out by Sapienza Archaeological Expedition in 2003^24,25^. This Fortress, bordering the West Gate and the adjacent city walls, is a rectangular building erected at the mid of the VI century and then, after Dionysios’ attack in 397 BC, renovated and dedicated to cult activities. The nail after the excavation was immediately set into a sealed box that stabilized the humidity (max 50%) at temperature s between 10 and 25 °C until it was brought to the lab.

The guidelines for the selection of these analytical methods are dictated by the necessity to get the maximum information on the whole artefact with the minimum sampling.

**Figure 1:**
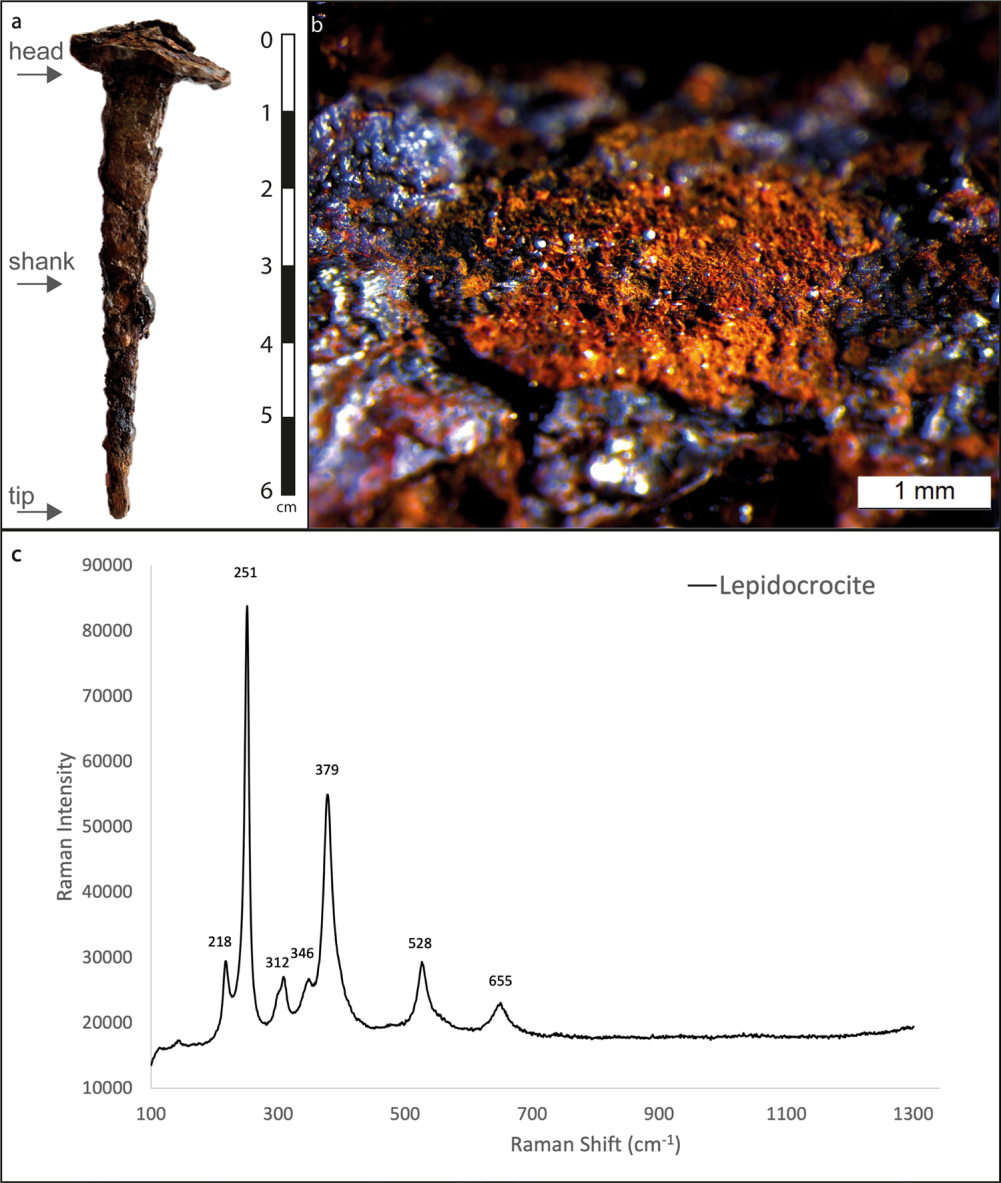
Mineralogical composition of nail patina. (**a**) Macroscopic view of the nail MF.03.53 (**b**) Optical microphotograph of reddish-brown patina and (**c**) Raman spectra of Lepidocrocite.

We started with a characterization of the external surface of the artefact, then exploring its interior both through a destructive cross-section of the tip (Fig. 1a) and through numerous 2D virtual tomographies of the entire sample, which allowed us to greatly increase the performance of the traditional analyses with a minimal sampling processing. Optical microscopy observations and micro-Raman spectroscopy were performed to investigate the external mineralogy of the patina. Chemical composition analyses by SEM-EDS on cross-section collected from the tip of the nail were performed to study microstructure of corrosion products, the nature of exogenous and slag inclusions. Moreover, the capabilities of XRM are employed to explore the corrosion propagation and verify the presence and the thickness of metal core in the entire object, monitoring the variations in density between different phases of the artefact. Indeed, sub-micron X-ray microscope ZEISS Xradia Versa 610 overcomes the limits of X-ray computed tomography (CT), offering a setup that enables non-destructive, multi-length scale visualization with an imaging field of view range from tens of millimeters down to tens of micrometers, and a true spatial resolution of 500 nm^21^.

Nails play a vital role in most construction contexts (e.g., ships, houses, sheds, boxes, coffins, doors, roofs, carts, sledges, etc.) and represent useful markers of technological advancements. In most excavation contexts, these functional artefacts are the only remaining record of wooden structures and, therefore, often have a pivotal role in archaeological arguments. The recording and interpretation of their function and manufacturing is thus of considerable importance for the reconstruction of ancient population lifeways.

This work aims to investigate the evolution of the corrosion process into a nail, for which a proper consideration must be given when studying the mechanical property of the object (i.e., strength, stiffness, durability) and its original microstructure that enable crucial information about manufacturing and performing features of the nail.

## Results and discussion

### Mineralogical composition of the external patina

Figure 1a shows a photographic image of the nail with inventory number MF.03.53. The surface of the nail was investigated with a Leica microscope M 125C equipped with a digital camera revealing a reddish-brown coarse crust that covered the artefact (Fig. 1b). Micro-Raman spectroscopy revealed that this rust layer evolved toward lepidocrocite (γ-FeO(OH), as revealed by the characteristic bands identified at 218, 251, 312, 346, 379, 528, 655 cm^−1^(Fig. 1c).

This mineral phase precipitates in slow aerial oxidation and hydrolysis of solid or aqueous iron compounds, at pH conditions greater than 3–5, as shown in Pourbaix diagrams of iron specific to chloride-containing aqueous media^26^.

### Microstructure and chemical composition in 2D real and virtual sections

A small fragment of the tip of the nail, as indicated in Fig. 1a, was carefully sampled and embedded in epoxy resin in order to prepare a “real” cross-section for SEM-EDS analysis.

**Figure 2:**
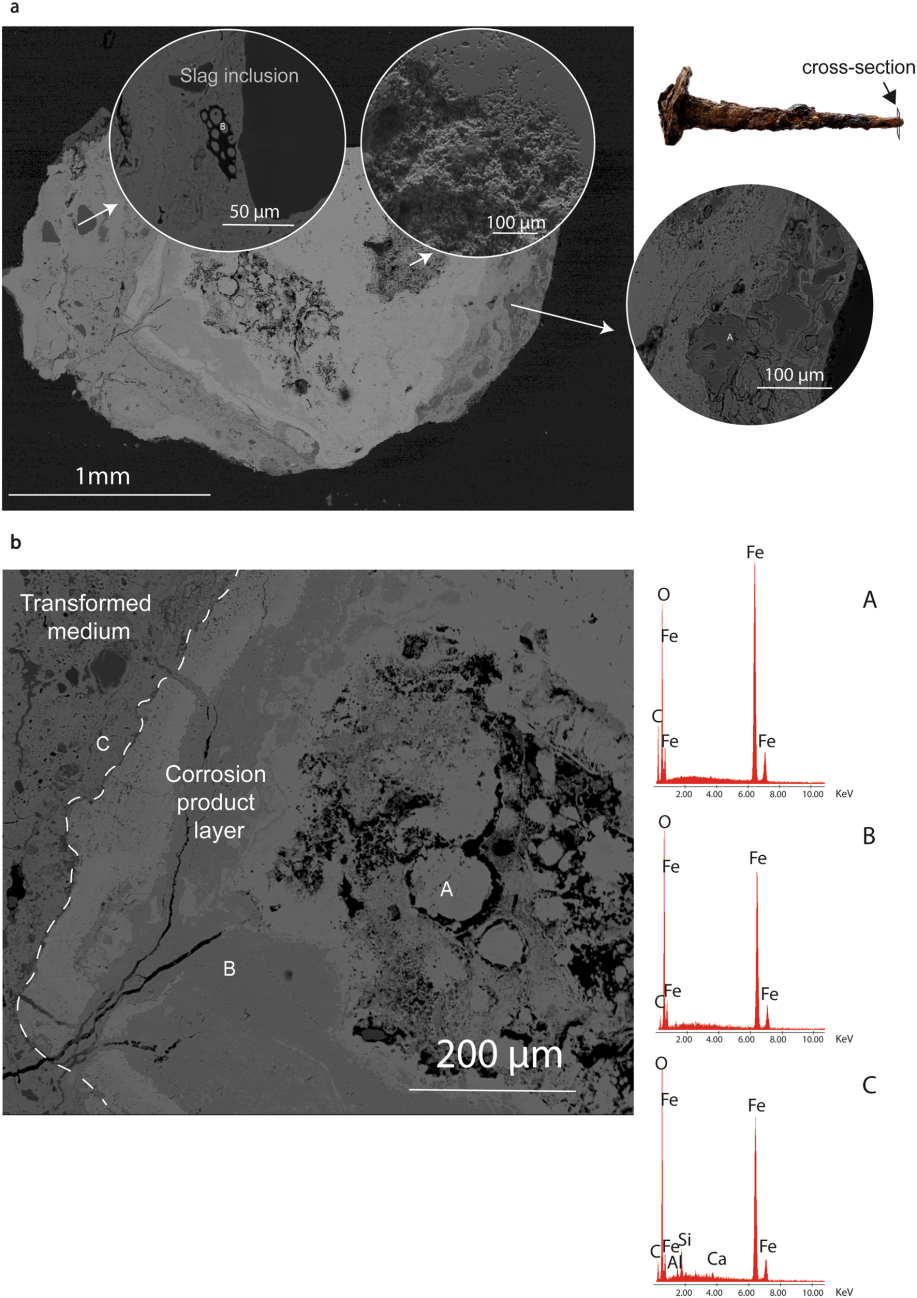
SEM–EDS images and spectra of the tip of the nail. (**a**) BSE image of the cross-section with the higher magnification images and spectra of slag inclusion, soil minerals and SE image of the crack. (**b**) Magnified view of the dense product layer (DPL).

As observed on the SEM image of Fig. 2, the shank of the nail is made up of a solid and quadrangular section, which has been forged with two-by-two opposing and orthogonal hammering. Thus, the corrosion layers and cracks are oriented in the direction of the length of the element.

Due to the stress generated by multiple forging operations in more directions, the core of the artifact is the most fragile and less compact. In Fig. 2a spongy structures and voids induced by the incomplete compaction of the iron-solid are visible during thermo-mechanical processing.

As shown in Fig. 2b, two corrosion layers are identified in iron nails as follows: dense product layer (DPL) composed of corroded iron phases and transformed media (TM), where the precipitated iron corrosion phases coexist with some soil elements (Ca, Si, Al) and slag inclusions, but no track of uncorroded metallic core is detected. The DPL shows two substructures, consisting of a light-area of magnetite (EDS spot A) and a slightly dark matrix of goethite (EDS spot B) (see also Supplementary Figs. S1 and S2). The TM layer, instead, is characterized by the presence of goethite and lepidocrocite (see also Supplementary Fig. S3).

The occurrence of magnetite, goethite and lepidocrocite and the absence of chloride phases suggest a low Cl^−^ activity in alkaline media, typical of the Lagoon-like system of Motya, as reported in^27^.

**Figure 3:**
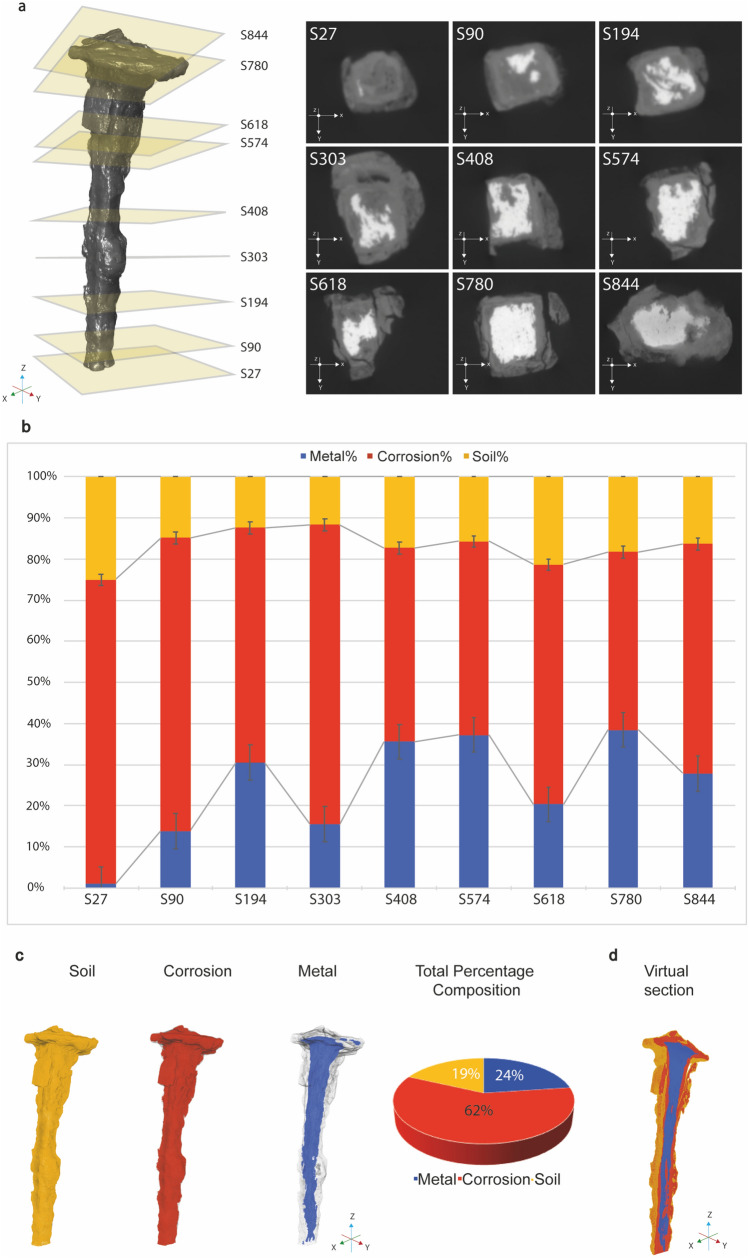
Low-resolution scans and 3D reconstruction of the nail. (**a**) On the left, the exact position of each virtual cross section in the nail shown on the right. (**b**) Histogram quantifying percentages of metal, corrosion and soil per each slice shown in a. (**c**) 3D reconstruction of the nail and segmentation of different layers: yellow—soil, red—corrosion layer, blue—metal; on the right, pie chart indicating the total percentages composition resulted by imaging segmentation. (**d**) Sagittal section of the 3D reconstruction showing the internal region of one half of the nail.

The degree of corrosion and the distribution of the corrosion products precipitated in the DPL and TM of the nail were further investigated by visual analysis with XRM. By non-destructive 3D imaging, nine virtual x–y cross-sections of the nail have been selected and shown in Fig. 3a. The exact position of each nail slice is indicated on the left of the image.

The grey-level scale of the 2D tomographies is related to the absorption coefficient, i.e. density value, of each region of the artefact: light grey corresponds to the metal core remaining, medium grey corresponds to corrosion layers and dark grey represents cracks and soil inclusions.

Several features are evident in the visualization of the 2D cross-sections. Metal core consistently does not appear until slice 90. As it progresses getting closer to the nail head, the metal core, which appears as light grey, increases in volume and becomes more compact, suggesting that the tip has corroded away, whereas the head and upper part of the shank still remain.

The corresponding percentages of each layer are shown per slice in the graph (Fig. 3b), where the amount of metal core remaining is measured.

The slices between 408 and 574, which correspond to the centre of the nail shank, have an iron content of 36–37%, whereas the slices 303 and 618 contain less metallic iron (16–20%). These sections are found in correspondence of reddish-brown clusters of corrosion products.

The lowest percentage of the metal core is recorded at the tip; conversely the higher iron content (39%) is noticed in slice 780, just below the nail head, and shows a more compact and regular metal section, suggesting isotropic pressure on all four sides. The head of the nail, on the other hand, has a metal iron content of about 28%. The main trend observed in Fig. 3b shows a significant uncorroded metal core in the nail head, while the tip is deeply corroded; however, some fluctuations are observed in the shank (e.g. slice 618), probably due to local stressed areas.

It was frequently observed that the head of the nail was still in good condition, while its body was heavily transformed into iron-rich rust^28^. This phenomenon suggests that corrosion was the result of moisture from the environment in contact with soils. It is possible that the nail was mounted vertically in the atmosphere where the bottom of the nail corroded faster than the top of the nail.

### Description of the 3D morphology

The morphology of the entire iron nail obtained *via* low-resolution X-ray scan is illustrated in Fig. 3c and Supplementary Movie 1.

To investigate how corrosion present in the sample varies spatially, a segmentation procedure was performed. According to the X-ray absorption coefficients of the main elements present in the sample and in relationship with the chemical composition previously analysed, we could differentiate the metal (blue), corrosion (red) and soil (yellow) volumes (Fig. 3c).

As shown by 3D model, the layer of soil perfectly follows the profile of the nail, distributing itself evenly over it and covering it like a thin “sock”; the corrosion products give the actual shape and dimensions to the object, resulting by the volume expansion during transformation of iron metal to iron oxy-hydroxides. The small nucleus of iron, which appeared in blue, indicated the loss as a result of corrosion.

A uniform corrosion from outer inwards towards the core of the nail was observed.

Generally, the metal core occupies only 24% of the total volume of the product, while about 62% has been transformed into rust and 19% is soil.

Sagittal cut of the 3D reconstruction, displaying the internal region of one half of the nail, shows that the head and upper part of the shank were very compact, whereas along the tip the percentage volume of the metal decreases (Fig. 3d).

### Microscale visualization of iron corrosion products on XRM experiments

**Figure 4:**
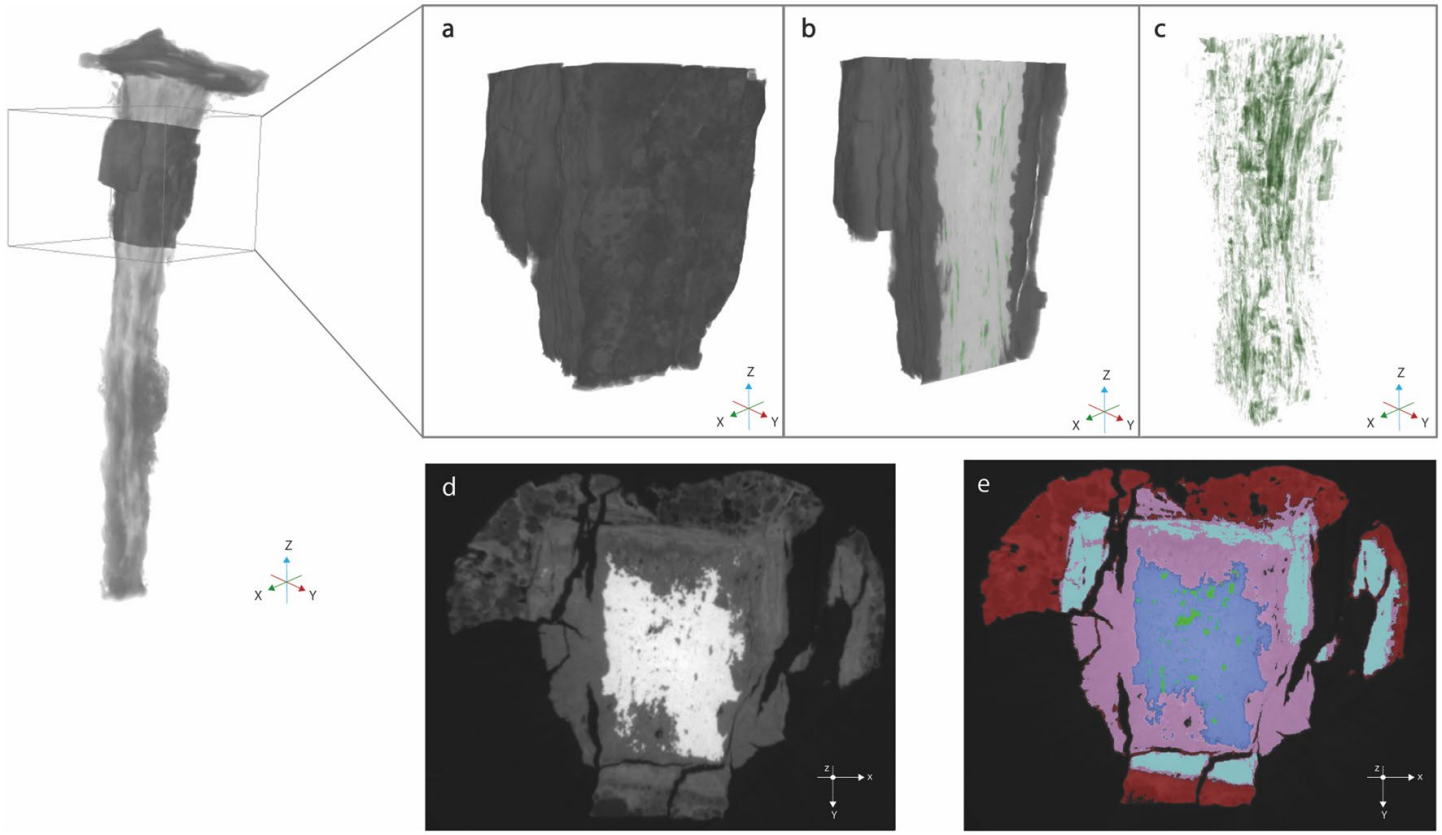
High-resolution scans of a selected volume of the nails. (**a**) 3D rendering of analysed section (**b**) Sagittal cut of the 3D reconstruction, showing parallel structural discontinuities (**c**) 3D segmentation of structural discontinuities (green) (**d**) 2D slice, showing microstructure detail and corrosion stratigraphy in grayscale value (**e**) Segmented 2D slice in false colors: metal in blue, DPL in pink (goethite) and turquoise (magnetite) and TM (red).

Although a low-resolution but large-field XRM scans is suitable to obtain a general overview of the nail understanding the phase structure and distribution, it is also necessary to run higher-resolution XRM scans of a selected volume of the nail (Fig. 4a) in order to study the stratigraphy of corrosion products and structural breaks in more detail (Supplementary Movie 2). Several structural discontinuities (green) have been observed in the inner metal core (blue) (Fig. 4b,c). High resolution scans revealed that most of the structural breaks are parallel to the metal/corrosion products interface, suggesting an origin from a lateral hammering during the forging phase and not during the nailing.

2D slice of Fig. 4d,e shows a further differentiation between the iron corrosion phases based on their grey-scale value and false colors. These layers and sub-layers are designated as goethite (pink) and magnetite (turquoise), according to the literature^29^. Compared with low-resolution scans, some veins composed of iron oxide (maghemite or/and magnetite) can now be observed in the dense product layer, not connected to the metal core.

First, at the metal/DPL interface, iron oxidizes and generates magnetite. With long-term burial, the corrosion front progresses and forms iron oxyhydroxides (goethite) in which a magnetite strip is present as a presumed trace of the initial layer. Indeed, the main phase observed at the interface between the metal and the rust layer is goethite. The formation of the TM, in red, could be explained by the dissolution of iron and reprecipitation of iron corrosion/oxidation products over the surface of the object, perhaps over the original surface^27,30–33^.

Goethite and lepidocrocite, detected by Micro-Raman technique in the TM layer, are difficult to discriminate on the basis of their attenuation coefficients as the two polymorphs have the same density. For this reason, we generically indicate the TM layer as a single corrosion phase designed with red color. Different grain orientation of the iron in the nail also played a role on the rate of corrosion. Furthermore, intergranular corrosion was observed in the metal core via X-ray tomography.

### Advantages and drawbacks of XRM over conventional techniques

In this research the preliminary investigation of the corrosion system of an iron nail was performed by traditional techniques. Micro-Raman spectroscopy allows us to verify an active corrosion on the external patina as confirmed by the presence of red-brown clusters of lepidocrocite. SEM imaging shows the lack of a metal core in the tip of the nail and the occurrence of corroded iron phases in the DPL and TM. Slag inclusions and quartz particles are embedded in goethite matrix. In order to evaluate if the section studied was representative of the entire sample, we perform a low-resolution scan of the nail with XRM. The challenge is to extract as much useful information as possible from the 3D visualization and image segmentation about the spatial distributions of phases in iron-based archeological nail. The main advantage is the ability to create virtual sections without cutting the sample. In particular, XRM workflow resulted an appropriate technique for: (1) exploring the stratigraphy of the sample without any invasive sample preparation as in traditional techniques, (2) investigating the internal structure of the artefact such as structural discontinuities to shed light on the manufacturing technique, (3) estimating the ratio of phases in each virtual cross section through the whole object.

In this way, we have demonstrated the existence of metal core in the shank and the head of the iron nail. However, the low-resolution scan represents a limiting factor for the identification of the corrosion sub-layers.

Thus, to tackle the complete characterization of the virtual section which includes the uncorroded metal, we worked also with high-resolution scan, covering all length scales from micrometric resolution to centimeters. Combining SEM-EDS data with XRM images of the entire nail the evolution of internal nail corrosion can be tracked.

The information provided by the tomography about the state of conservation of an archaeological artefact have two interesting applications: from museum and conservative point of view, a periodic inspection of sample could help to study joins, hidden cracks and repairs or earlier restoration attempts in a non-destructive manner. On the other hand, from a diagnostic point of view, this analysis could represent a preliminary check of the whole object to address the sampling of the section in order to investigate a specific area of interest, when it is possible. An important limitation of XRM scan is represented by the impossibility to obtain precise chemical information about the sample components.

## Methods

Micro-Raman spectroscopy: was used on polished cross-sections to determine the mineralogical composition of phases. Micro-Raman analyses were performed at room temperature using a Renishaw RM2000 equipped with a Peltier-cooled charge-coupled device camera in conjunction with a Leica optical microscope with 10×, 20×, 50× and 100× objectives. Measurements were performed using the 50× objective (laser spot diameter of about 1 μm) and the 785 nm line of a laser diode. Two edge filters blocked the Rayleigh-scattered light below 100 cm^−1^. For this reason, the study of ultra-low wavenumber Raman spectra in the region < 100 cm^−1^ is overlooked. In order to avoid damaging the patina and to prevent the fluorescence from covering the Raman signal, the laser power was lowered. No baseline was subtracted from the recorded spectra. The spectra obtained were compared with GRAMS spectroscopy software and databases available in the literature.

SEM-EDS investigations: were carried out by FEI-Quanta 400 instrument, equipped with X-ray energy-dispersive spectroscopy (Department of Earth Sciences, Sapienza University, Rome, Italy). SEM imaging was collected both in secondary electron (SE) and back scattered electron (BSE) modes. Energy-dispersive X-ray spectroscopy (EDS) spectra and X-ray maps were also used to show the distribution of the elements through the samples. Sample was coated with a thin layer of carbon in order to avoid charging effects. EDS spectra were collected at a 25 kV and a pressure in the analysis chamber of 10^−4^ Pa.

X-ray microscopy: was performed using a laboratory X-ray microscope (Zeiss, Xradia Versa 610) at Sapienza Nanoscience & Nanotechnology Laboratories (SNN-Lab) of the Research Center on Nanotechnology Applied to Engineering (CNIS) of Sapienza University. When scanned, the nail was fixed on the sample stage between the X-ray source and detector by a clamp sample holder. The sample stage can rotate around a fixed axis to ensure a full 360° scan. For the low-resolution scan the sample-to-detector distance was set to 26 mm, and source-to-sample distance was 221 mm. The voltage and current of the X-ray beam were 150 kV and 152 μA, respectively, and the power was set to 23 W. Scans were performed from 0° to 360° using a 0.4× objective and the exposure time for each projection was set to 2 s. A total scanning time of circa 2 h was required. Acquired images were obtained with a pixel size of 61.31 μm and binned (2 × 2 × 2). For the high-resolution scan sample-to-detector distance was set to 95.6 mm, and the source-to-sample distance was set to 25.6 mm. The voltage and current of the X-ray beam were 150 kV and 152 μA, respectively, and the power was set to 23 W. Scans were performed from 0° to 360° using a 0.4× objective and the exposure time during each projection was set to 1 s. A total scanning time of circa 2.5 h was required. The stacks of grayscale images were imported into the Reconstructor Scout-and-Scan software to manually find the center shift and the beam hardening constant. The image processing step was carried out using the Dragonfly Pro software.

3D models reconstruction: 3D models were reconstructed using the Reconstructor module of Zeiss Scout and Scan control softwareScout&Scan Control System Reconstructor software - Zeiss (Version 16.1.13038.43550) by which reconstruction parameter was identified. Regarding the low-resolution scan, the Center Shift was manually set at − 0.800, the Beam Hardening Constant was set manually at 0.09 and 1601 projections were selected to reconstruct the 3D model. Concerning the high-resolution scan, the Center Shift was manually set at 1.200, the Beam Hardening Constant was manually set at 0.07, and 1601 projections were used to reconstruct the 3D model. In both cases, at the end of the process a TIFF stack was exported and subsequently imported in Dragonfly Pro software (Version 2020.1 Build 797, Object Research System) for post processing. Dragonfly Pro was used to visualize the iron nail excluding the signal coming from the air around the sample through the window leveling tab.

## Supplementary Information

Supplementary Information.

Supplementary Video 1.

Supplementary Video 2.
