# Supplementary material for: A comprehensive strategy for exploring corrosion in iron-based artefacts through advanced Multiscale X-ray Microscopy
Source: Sci Rep. 2022 Apr 12;12:6125. doi: 10.1038/s41598-022-10151-w (PMC9005549; doi:10.1038/s41598-022-10151-w)
Supplement: Supplementary file 1 — Supplementary Information. [file 41598_2022_10151_MOESM1_ESM.pdf]

A comprehensive strategy for exploring corrosion in iron-based artefacts through advanced Multiscale X-ray Microscopy

Martina Bernabale, Flavio Cognigni, Lorenzo Nigro, Marco Rossi, Tilde de Caro, Caterina De Vito

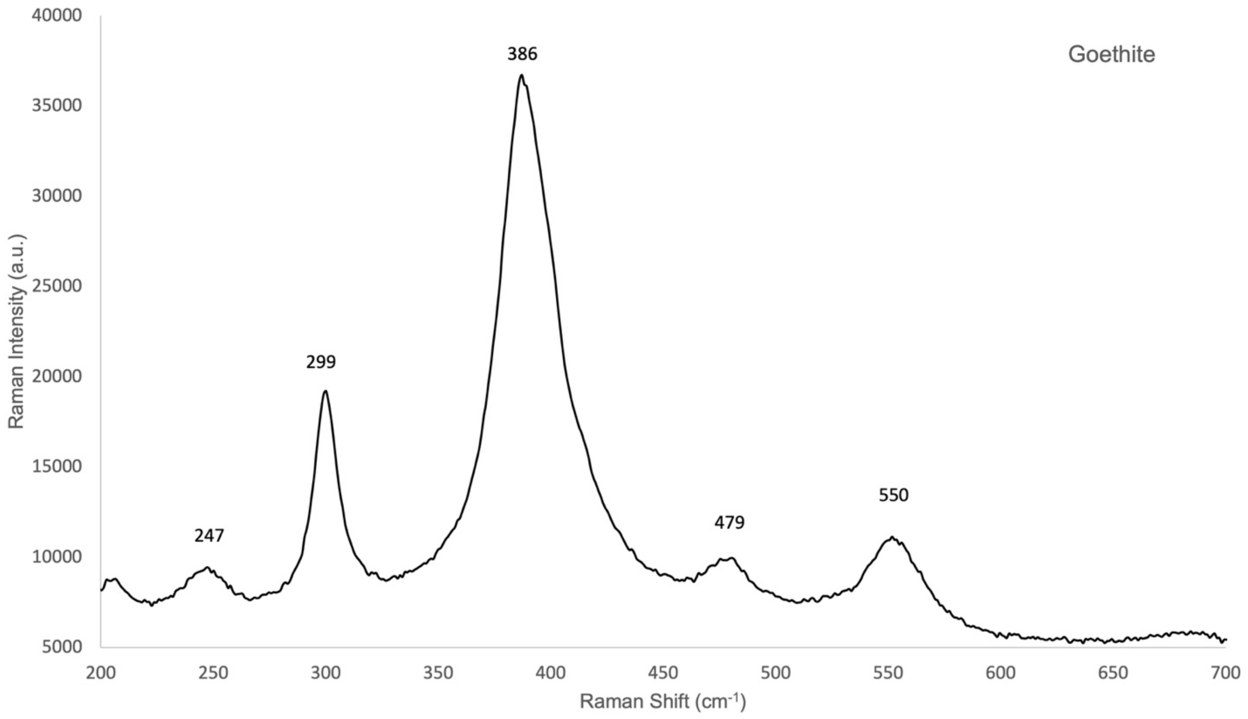

Supplementary Figure S1. Raman spectrum of corrosion product on nail section. Raman spectrum of goethite in DPL

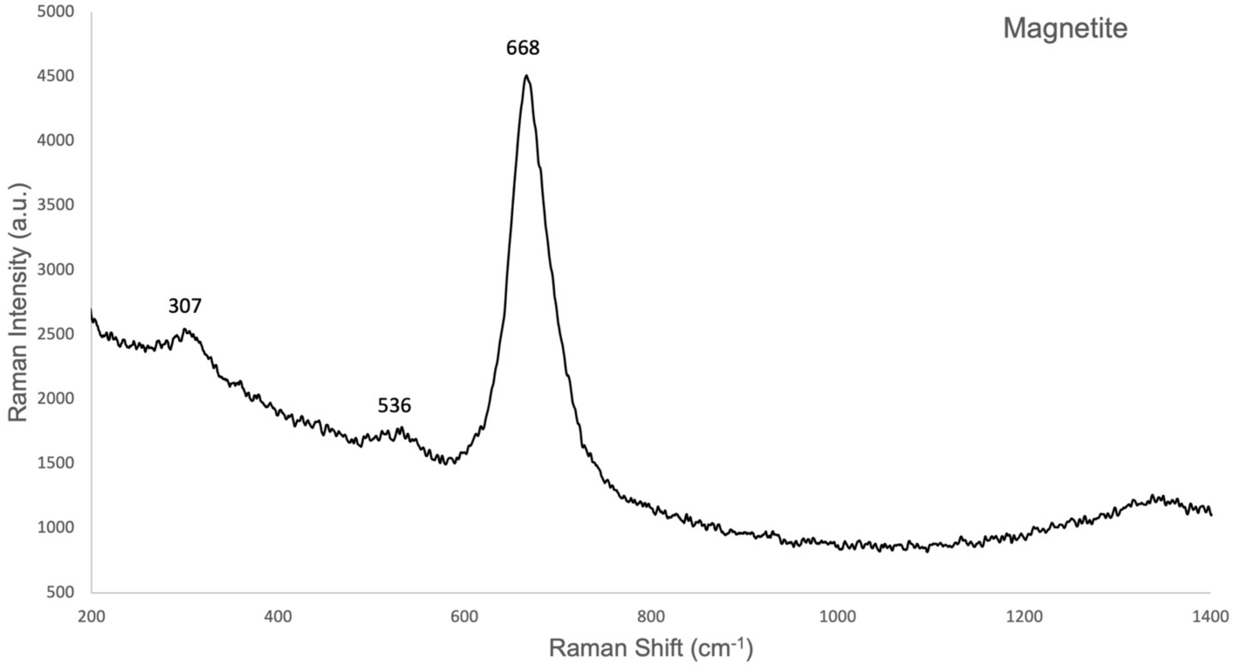

Supplementary Figure S2. Raman spectrum of corrosion product on nail section. Raman spectrum of magnetite in DPL

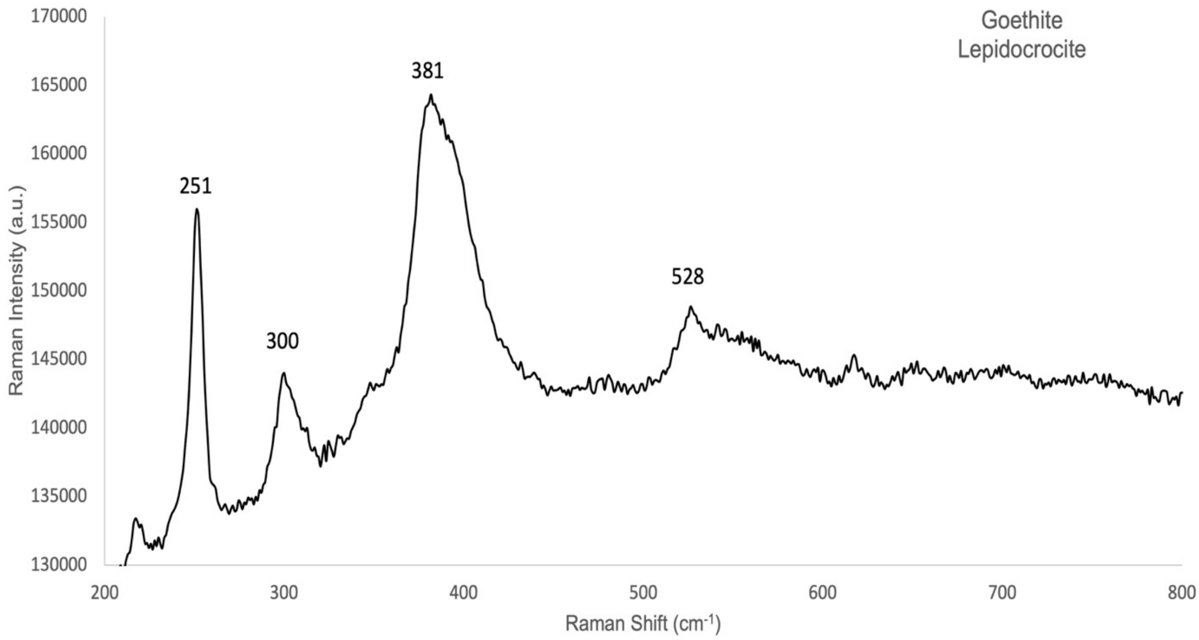

Supplementary Figure S3. Raman spectrum of corrosion product on nail section. Raman spectra of goethite and lepidocrocite in TM

Supplementary Movie S1: 3D reconstruction of the iron nail *via* low-resolution X-ray scan.

Supplementary Movie S2: 3D reconstruction of a selected volume of the iron nail *via* high-resolution X-ray scan.
